# Recurrence of atrial fibrillation after cardiac surgery: long-term evidence from cardiac devices

**DOI:** 10.3389/fcvm.2025.1669461

**Published:** 2025-10-03

**Authors:** Tim Swinn, Marius Pezard-Snell, Lauren Brain, Gerasimos Dimitropoulos, Amardeep Dastidar, Eva Sammut, Palash Barman

**Affiliations:** ^1^ Department of Cardiology, North Bristol NHS Trust, Bristol, United Kingdom; ^2^ School of Medicine, University of Bristol, Bristol, United Kingdom; ^3^ Department of Cardiology, Bristol Heart Institute, Bristol, United Kingdom; ^4^ Department of Anaesthetics, Royal United Hospitals, Bath, United Kingdom; ^5^ Department of Cardiology, Royal Devon & Exeter NHS Trust, Exeter, United Kingdom

**Keywords:** atrial fibrillation, AF, subclinical, cardiac surgery, device-detected AF

## Abstract

**Introduction:**

Atrial fibrillation (AF) after cardiac surgery occurs in 20%–40% of cases and is associated with significant morbidity. Studies have shown an association with immediate postoperative AF (POAF) and recurrent AF; however, to our knowledge, no trials have used continuous electrogram monitoring data from cardiac devices (pacemakers or defibrillators) to assess the rate of long-term AF recurrence.

**Methods:**

Using institutional databases, we identified patients with a cardiac device who underwent coronary artery bypass grafting (CABG) and/or a procedure on their aortic valve (AV) or mitral valve (MV) between January 2011 and March 2020. POAF and comorbidities were assessed using the electronic patient record, and recurrent device-detected AF was defined as any episode of AF lasting >6 min on device check between 6 weeks and 18 months postoperatively.

**Results:**

POAF was associated with recurrent device-detected AF (*n* = 85, odds ratio 3.26, 95% confidence interval 1.19–8.97, *p* = 0.02). Age was an independent risk factor for developing POAF (*n* = 302, *p* = 0.033), and MV surgery had a higher rate of POAF than CABG alone (54% vs. 32%, *p* = 0.047) and AV surgery (54% vs. 34%, *p* = 0.048). Forty-four per cent of patients developing POAF were discharged on oral anticoagulation (OAC).

**Discussion:**

Postoperative is associated with a threefold greater odds of developing recurrent device-detected AF: it is not a simple transient phenomenon. Larger prospective studies are required to identify which patients would benefit from heart rhythm surveillance and whether OAC is an effective treatment in this patient group.

## 1 Introduction

Atrial fibrillation (AF) is a common postoperative complication of open cardiac surgery, with an incidence of 20%–40% (1). It is associated with increased early postoperative morbidity, including stroke, renal dysfunction, respiratory dysfunction, prolonged intensive care admissions, and early and late mortality (2–5). The majority of patients who experience postoperative AF (POAF) revert to sinus rhythm by discharge; therefore, POAF was previously considered a transient postoperative phenomenon. However, recent studies have shown an association between POAF and stroke over a 10-year follow-up period (1). A recent cohort study found that 33.1% of patients who developed AF for the first time during hospitalisation for non-cardiac surgery or medical illness experienced at least one further episode of AF lasting longer than 30 s within 12 months following admission, compared with 5.0% in a matched control group (6). This is despite all patients having reverted to sinus rhythm by discharge. A 2024 individual patient data meta-analysis included 185 participants from eight prospective trials who had undergone CABG or valve surgery, had developed POAF, and subsequently had an implantable loop recorder (ILR) inserted (7). Kaplan–Meier analysis demonstrated a 35.3% (27.6%–42.2%) chance of AF recurrence 18 months after surgery for patients who developed AF within 30 days of surgery. However, the majority of trials included in that meta-analysis lacked a control arm of matched patients who did not develop POAF and therefore were unable to assess the additional risk of recurrent AF that POAF conferred (8–14). One prospective observational trial using ILRs did find that early POAF (as defined by within 5 days of cardiac surgery) was associated with a significantly higher incidence of late POAF up to 36 months after surgery compared with patients who did not develop POAF [*n* = 79, hazard ratio (HR) 3.5, 95% confidence interval (CI) 1.7–7.1, *p* = 0.001] (15).

An opportunity to gain long-term continuous ECG monitoring without additional intervention presents itself in the form of cardiac devices, namely, a pacemaker (PPM) or implantable cardiac defibrillator (ICD). These devices may be inserted pre-surgery or in the immediate postoperative period due to complications such as heart block or ventricular arrhythmia. In addition to their therapeutic functions, these devices provide valuable monitoring information about arrhythmias and AF burden. This forms the premise of our study; we aimed to assess whether POAF after open cardiac surgery [CABG, aortic valve (AV) surgery, or mitral valve (MV) surgery] is associated with recurrent AF by using continuous monitoring provided by capable cardiac devices that had been inserted before cardiac surgery or up to 6 weeks postoperatively. Secondary outcomes included identifying risk factors for developing POAF and evaluating the incidence of device insertion by operation type.

## 2 Materials and methods

### 2.1 Data, inclusion criteria, and outcomes

We used an institutional cardiac surgical database containing all operations between January 2011 and March 2020 to identify patients who underwent coronary artery bypass grafting (CABG) or surgery on the AV or MV. Patients were grouped into three categories, i.e., “CABG-only”, “AV surgery ± CABG”, and “MV surgery ± CABG”. This database was merged with an electrophysiology database to identify patients who had undergone surgery and had a cardiac device capable of recording atrial activity *in situ* (dual chamber pacemakers, dual chamber implantable cardiac defibrillators, or cardiac resynchronisation devices). The inclusion and exclusion criteria are listed below.

Inclusion criteria:

1. CABG and/or surgery on the mitral or AV between January 2011 and March 2020.
2. A pre-existing cardiac device or one inserted <6 weeks postoperatively.
3. Adequate data to assess the presence or absence of postoperative atrial fibrillation (at a minimum via a discharge letter).
4. Additional criterion for the recurrent AF arm: at least one device check result available >6 weeks postoperatively.

Exclusion criteria:

1. Pre-existing diagnosis of AF, AF on preoperative ECG, or AF on any preoperative device check.
2. Congenital heart disease.
3. Surgery on multiple heart valves.
4. Preoperative AF ablation or intra-operative surgical AF ablation.
5. Died <6 weeks postoperatively.
6. Inadequate information in the immediate postoperative period.

Clinical information was gathered from the electronic patient record (EPR). Demographic data including age, gender and body mass index, and presence of comorbidities including hypertension, diabetes mellitus (type 1 or type 2), preoperative left ventricular ejection fraction (LVEF), previous stroke, peripheral vascular disease, and hypercholesterolaemia were recorded. The EPR and electronic observations were used to assess whether a patient had developed POAF after surgery and prior to discharge. If a patient had two operations in the period, only their first operation was included, and device follow-up was stopped at the time of the second operation.

The primary outcome was recurrent device-detected AF, as defined by at least one episode of high atrial rate (confirmed as AF on atrial electrogram) lasting greater than 6 min on any post-surgical device check beyond 6 weeks post-operation. Device checks performed within 6 weeks were excluded. The 6 min duration of AF was adopted from the major randomised controlled trials (RCTs) investigating the use of anticoagulation in patients with device-detected subclinical atrial fibrillation (16–18). Subclinical AF episodes lasting longer than 6 min are associated with a higher incidence of ischaemic stroke or systemic embolism (18). All available device checks between 6 weeks and 18 months post-surgery were reviewed.

### 2.2 Statistics

IBM SPSS Statistics Software (version 29.0.2.0) was used for statistical analysis. For comparison of characteristics between groups that developed POAF and those that did not, the independent *t*-test or chi-square tests were used (depending on the nature of the independent variable). Binary logistic regression analysis was performed in both the POAF and recurrent AF arms of the study. This analysis was conducted initially using univariable binary logistic regression to assess for potential associations, and variables with *p* < 0.10 were included in the subsequent multivariable binary logistic regression. Independent variables were considered to have a statistically significant effect if *p* < 0.05 after multivariable binary logistic regression. Effect sizes are reported as odds ratios with 95% confidence intervals.

### 2.3 Sample size justification

This was a retrospective observational study using an institutional database, which determined the available sample size. An *a priori* sample size calculation was therefore not applicable; however, we aimed to maximise statistical power by including all consecutive patients within the stated time frame for whom we had adequate data. The recurrent AF arm sample size was limited by patients who underwent device follow-up at other centres, as data from these centres were not accessible.

## 3 Results

### 3.1 Devices

**Figure 1:**
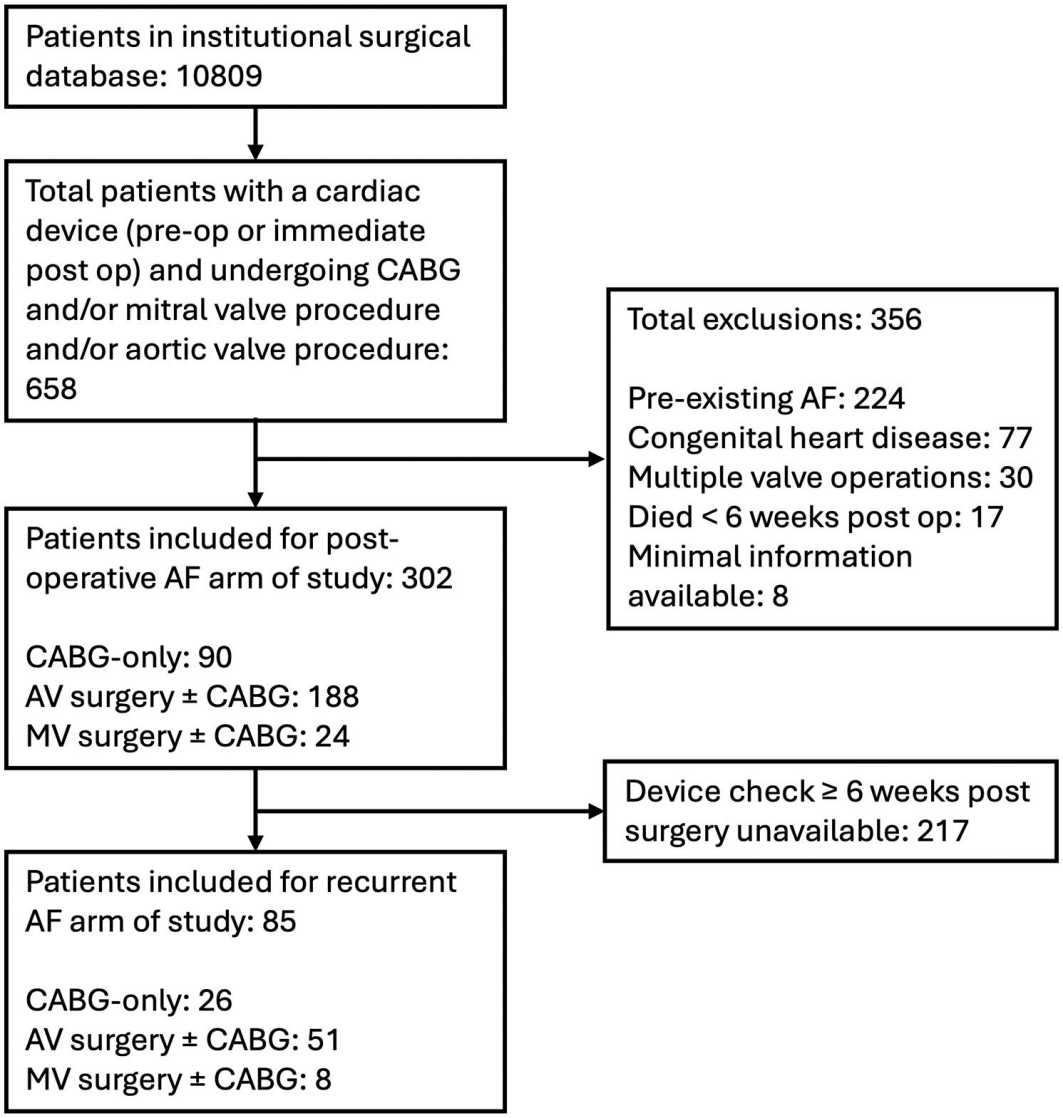
Consort diagram showing breakdown of patients included in study. CABG, coronary artery bypass grafting; AF, atrial fibrillation; AV, aortic valve; MV, mitral valve.

A total of 10,809 operations were conducted between January 2011 and March 2020 (CABG-only, 6,470; AV surgery ± CABG, 3,244; MV surgery ± CABG, 1,095). After selecting for patients with devices inserted preoperatively or up to 6 weeks post-op and applying exclusion criteria, 302 patients were included for the devices and POAF arms of the study (CABG-only, 90; AV surgery ± CABG, 188; MV surgery ± CABG, 24). Figure 1 presents a CONSORT diagram showing the number of patients included in each arm of the trial and reasons for exclusion. Of the 302 included patients, 74 (25%) were female, and the mean age was 69 [standard deviation (SD) 10.1].

**Figure 2:**
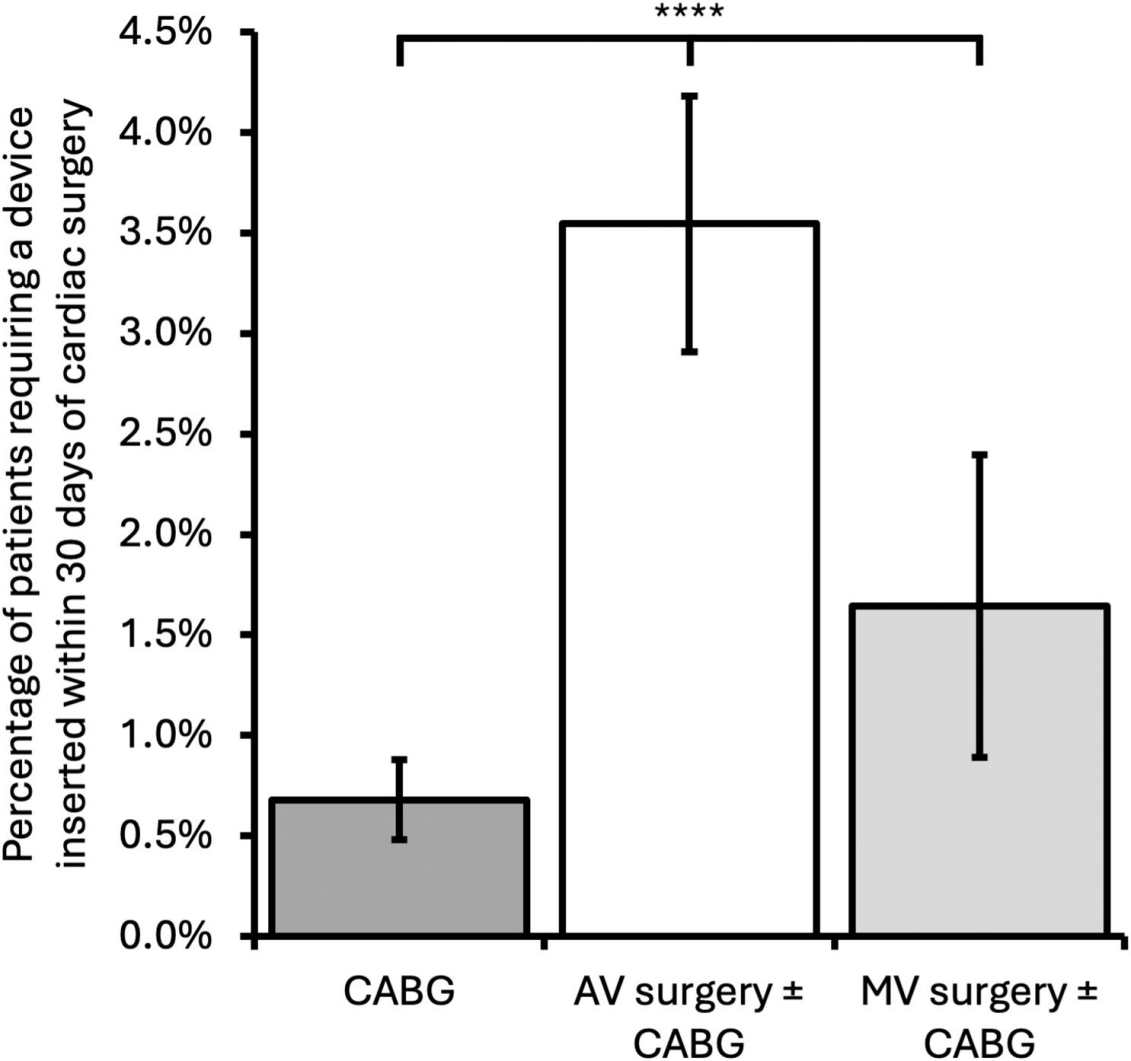
Incidence of patients requiring a cardiac device inserted within 30 days of cardiac surgery comparing surgery type. Error bars represent 95% confidence intervals. CABG, coronary artery bypass grafting; AV, aortic valve; MV, mitral valve; ****p < 0.0001.

AV surgery was associated with a significantly higher incidence of cardiac device insertion ≤30 days post-operation than CABG-only or MV surgery ± CABG [CABG-only, 44/6,470 operations (0.68%); AV surgery ± CABG, 115/3,244 (3.5%); MV surgery ± CABG, 18/1,095 (1.64%); *p* < 0.0001, Figure 2]. This finding is consistent with previous studies demonstrating a significantly higher incidence of postoperative heart block following AV surgery (19).

### 3.2 Postoperative AF

**Figure 3:**
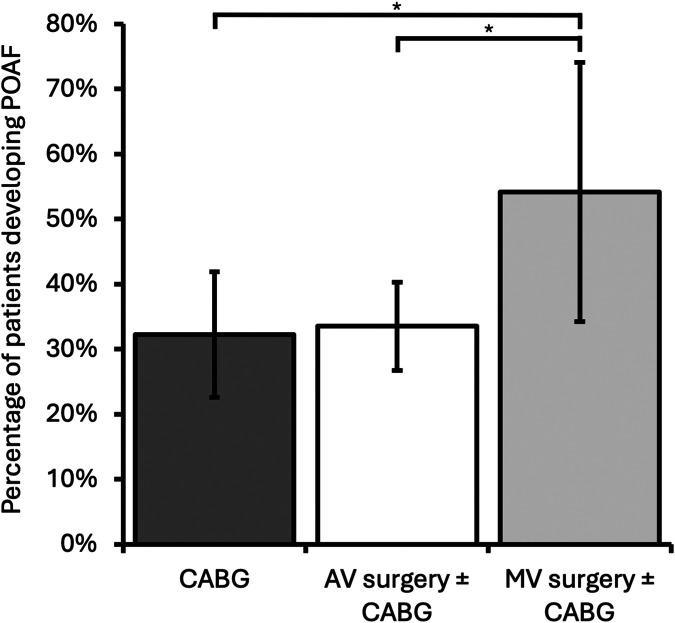
Incidence of patients developing POAF by operation category. Mitral valve ± CABG had a significantly higher incidence of POAF than both CABG-only and AV ± CABG operations. Error bars represent 95% confidence intervals. CABG, coronary artery bypass grafting; AV, aortic valve; MV, mitral valve; *p < 0.05.

A total of 302 patients were included for this arm of the study (Figure 1), of whom 105 (35%) developed POAF. MV surgery was associated with a significantly higher incidence of POAF compared with both CABG-only and AV surgery ± CABG [MV surgery ± CABG, 13/24 (54%); CABG-only, 29/90 (32%); AV surgery ± CABG, 63/188 (34%); CABG-only vs. MV surgery ± CABG *p* = 0.048, AV surgery ± CABG vs. MV surgery ± CABG *p* = 0.047, CABG-only vs. AV surgery ± CABG *p* > 0.05]. Development of POAF by operation type is shown in Figure 3*.* Age was associated with the development of POAF after both univariable and multivariable analysis [POAF mean age 71 years old (SD: 9.0), no POAF mean age 68 years old (SD: 10.5), *p* = 0.033]. No other components of the CHA_2_DS_2_-VASc score (heart failure, hypertension, diabetes mellitus, stroke/transient ischaemic attack, peripheral vascular disease, and gender) were associated with a statistically significant change in incidence of POAF, nor was the total CHA_2_DS_2_-VASc score [POAF mean CHA_2_DS_2_-VASc, 2.7 (SD 1.4); no POAF mean CHA_2_DS_2_-VASc, 2.7 (SD 1.3), *p* > 0.05]. BMI was similar in both groups [POAF mean BMI, 28.7 (SD 5.3); no POAF mean BMI, 27.9 (SD 4.6)]. The results are summarised in Table 1*.*

**Table 1:** Characteristics of patients in the POAF and no POAF groups. Only age remained statistically significant after multivariable analysis.

Demographics	Total	POAF	No POAF	*p*-value
		*n* (%)	*n* (%)	
*n*	302	105 (34.8%)	197 (65.2%)	
Operation type				
CABG	90	29 (27.6%)	61 (31.0%)	*N.S.*
AV surgery ± CABG	188	63 (60%)	125 (63.5%)	*N.S.*
MV surgery ± CABG	24	13 (12.4%)	11 (5.6%)	*N.S.*
CHA_2_DS_2_-VASc components				
LVEF < 50%	114	36 (34.3%)	78 (39.6%)	*N.S.*
Hypertension	212	72 (68.6%)	140 (71.1%)	*N.S.*
Age ± SD		71 ± 9.0	68 ± 10.5	*0.033*
Diabetes	56	19 (18.1%)	37 (18.8%)	*N.S.*
Stroke/TIA	34	15 (14.3%)	19 (18.1%)	
Vascular disease	104	32 (30.5%)	72 (36.5%)	*N.S.*
Female	74	23 (21.9%)	51 (25.9%)	*N.S.*
Total CHA_2_DS_2_-VASc ± SD		2.7 ± 1.4	2.7 ± 1.3	*N.S.*
BMI ± SD		28.7 ± 5.3	27.9 ± 4.6	

CABG, coronary artery bypass grafting; AV, aortic valve; MV, mitral valve; LVEF, left ventricular ejection fraction; SD, standard deviation; TIA, transient ischaemic attack; N.S., not significant (*p* > 0.05).

After excluding patients with alternative indications for oral anticoagulation (OAC) (mechanical heart valves, pulmonary or venous thromboembolism), 40 of the remaining 91 POAF patients (44%) were discharged on OAC.

### 3.3 Recurrent device-detected AF

**Figure 4:**
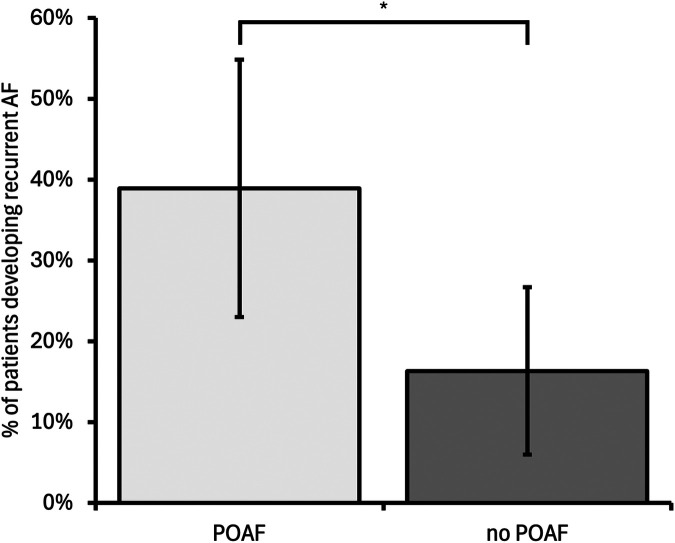
Incidence of recurrent device-detected AF for patients who developed POAF compared with those who did not develop POAF. POAF was associated with a higher incidence of recurrent AF compared with no POAF [odds ratio 3.26 (95% confidence interval 1.19–8.97), *p* = 0.022]. Error bars represent 95% confidence intervals. POAF, postoperative AF; *p < 0.05.

Device follow-up data beyond 6 weeks post-operation were available for 85/302 patients (28%, Figure 1). The median follow-up duration was 435 days (interquartile range: 171 days). This was similar for both patients who did and did not develop recurrent device-detected AF. POAF was associated with a significantly increased rate of recurrent AF [POAF patients developing recurrent AF, 14/36 (38.8%); patients without POAF developing recurrent AF, 8/49 (16.3%); odds ratio (OR) 3.26, 95% CI 1.19–8.97, *p* = 0.02, Figure 4]. Female gender and age were included in multivariable analysis after univariable analysis yielded *p*-values < 0.10; however, they did not remain significantly associated with recurrent AF after multivariable analysis. None of the components of CHA_2_DS_2_-VASc, total CHA_2_DS_2_-VASc, or BMI were associated with recurrent AF. The results are summarised in Table 2*.*

**Table 2:** Characteristics of patients in recurrent device-detected AF and no recurrent AF groups. Only POAF was significantly associated with the development of recurrent AF (OR 3.26, 95% CI 1.19–8.97). *p*-values for hypertension and gender are included as these variables were added to the multivariable analysis but did not reach the significance threshold of *p* < 0.05.

Demographics	Total	Recurrent AF	No recurrent AF	*p*-value
		*n* (%)	*n* (%)	
*n*	85	22 (25.8%)	63 (74.1%)	
Median follow-up duration in days (IQR)		413 (399)	435 (113)	*N.S.*
BMI ± SD		29.5 ± 3.6	30.1 ± 6.2	*N.S.*
POAF	36	14 (63.6%)	22 (34.9%)	*0.022*
Operation type				
CABG	26	5 (22.7%)	21 (33.3%)	*N.S.*
AV surgery ± CABG	51	14 (63.6%)	37 (58.7%)	*N.S.*
MV surgery ± CABG	8	3 (13.6%)	5 (7.9%)	*N.S.*
CHA_2_DS_2_-VASc components				
LVEF < 50%	31	6 (27.3%)	25 (39.7%)	*N.S.*
Hypertension	62	19 (86.4%)	43 (68.3%)	*0.082*
Age ± SD		69 ± 6.9	65 ± 9.6	*N.S.*
Diabetes	13	4 (18.2%)	9 (14.2%)	*N.S.*
Stroke/TIA	30	3 (13.6%)	7 (11.1%)	*N.S.*
Vascular disease	10	8 (36.4%)	25 (30.7%)	*N.S.*
Female	33	6 (27.3%)	24 (38.1%)	*0.088*
Total CHA_2_DS_2_-VASc ± SD		2.7 ± 1.1	2.5 ± 1.3	*N.S.*

POAF, postoperative atrial fibrillation; IQR, interquartile range; CABG, coronary artery bypass grafting; AV, aortic valve; MV, mitral valve; LVEF, left ventricular ejection fraction; SD, standard deviation; TIA, transient ischaemic attack; N.S., not significant (*p* > 0.05).

## 4 Discussion

To the best of our knowledge, this is the first study to use existing cardiac devices to directly measure AF in the postoperative setting. Our key findings include demonstrating that developing POAF after cardiac surgery (CABG, AV or MV procedures) is an independent predictor of developing clinically significant recurrent device-detected AF up to 18 months after the operation (OR 3.26, 95% CI 1.19–8.97, *p* = 0.02, Figure 4), and age is an independent predictor of developing POAF. The association of POAF with recurrent AF is an important finding and supports a growing body of evidence that provoked AF (after cardiac or non-cardiac surgery or acute hospital admission) is not simply a benign and transient phenomenon (1, 6). Our results support the hypothesis that chronic AF may underlie the higher incidence of stroke in patients who develop POAF (1).

Operation type and age were predictors of POAF. MV surgery ± CABG had a higher rate of POAF than CABG-only (54% vs. 32%, *p* = 0.047) and AV surgery ± CABG (54% vs. 34%, *p* = 0.048), as demonstrated in Figure 3. This finding is consistent with previous studies and likely reflects the proximity of the surgical site to the left atrium (20–24). In our study, age was an independent predictor of POAF (*p* = 0.033), but interestingly, other features of the CHA_2_DS_2_-VASc score did not reach statistical significance. This initially may not be surprising since the CHA_2_DS_2_-VASc score was developed as a model for the prediction of stroke risk in patients with clinical AF and was not designed as a predictor of POAF (25). Nonetheless, the risk factors have been shown to overlap, and a 2021 study by Burgos et al. (26) showed that all features of the CHA_2_DS_2_-VASc and total CHA_2_DS_2_-VASc score were associated with the development of POAF. Burgos et al. went further and proposed a new score (acronym: COM-AF), which had a higher area under the receiver operating characteristic curve (AUC) for predicting POAF [AUC 0.78 (95% CI 0.76–0.80)]. Components of COM-AF match those of CHA_2_DS_2_-VASc but exclude vascular disease. We did not find significant associations between these variables and POAF; however, this may be due to our sample size.

Best management of POAF remains unclear, and practice around anticoagulation varies between clinicians. Forty-four per cent of our POAF group were discharged on OAC (excluding those with alternative indications); however, not all patients with POAF will develop recurrent device-detected AF. Indeed, over 60% of the patients with POAF in our study did not develop recurrent AF, and one may assume these patients would receive a smaller benefit, or even no benefit of OAC, but still be exposed to the same bleeding risks (27). Evidence for OAC in the POAF setting is mostly from observational studies and has yielded conflicting results. This is demonstrated by two meta-analyses published in 2021. Neves et al. (27) and Fragão-Marques et al. (28) conducted meta-analyses of observational studies comparing long-term outcomes for POAF patients prescribed OAC vs. not prescribed OAC and reached opposing conclusions. Neves et al. (27) concluded that OAC was associated with reduced thromboembolic events in POAF patients post-cardiac surgery, but no such effect was seen after non-cardiac surgery. OAC was associated with increased bleeding in this study. Conversely, Fragão-Marques et al. (28) reported an association between OAC therapy and reduced all-cause mortality at 5 years for POAF patients, although this is likely to be noise, given they found no association between OAC therapy and reduced thromboembolism in POAF after cardiac surgery. The 2024 European Society of Cardiology (ESC) AF guidelines (29) include a section on provoked AF, specifically POAF. The ESC guidelines acknowledge the lack of high-quality evidence in this area but recommend considering long-term OAC for patients with POAF after cardiac and non-cardiac surgery at elevated thromboembolic risk (Class IIa recommendation, Level B evidence).

The broader topic of subclinical AF (outside of post-surgical patients) has become a hot topic in recent years, with two large-scale RCTs published in *The New England Journal of Medicine* in 2023 assessing the efficacy of OAC in subclinical device-detected AF, namely, NOAH-AFNET (16) and ARTESiA (17). Although initially conflicting results (NOAH-AFNET was prematurely terminated due to futility while ARTESiA found the OAC apixaban reduced stroke compared with aspirin), a subsequent meta-analysis including both trials has shown the results to be consistent (30). The meta-analysis concluded that OAC reduced stroke compared with placebo/aspirin (relative risk 0.68, 95% CI 0.50–0.92); however, there was an increase in major bleeding (relative risk 1.62, 95% CI 1.05–2.50). The 2024 ESC AF guidelines reflect this evidence and recommend that OAC therapy may be considered in subgroups of patients with asymptomatic device-detected subclinical AF who have high estimated stroke risk and an absence of major bleeding risk factors (Class IIb recommendation, Level B evidence) (29). Notably, the strength of this recommendation is lower than the Class Ia recommendation for anticoagulation in clinical AF.

Clinical AF has been shown to have higher morbidity than subclinical AF (31). The role for OAC in these patients is more established and likely to be of greater benefit than for patients with subclinical, device-detected AF. In our trial, data regarding clinical AF in the recurrent AF arm were not available due to the wide variety of healthcare settings to which a patient may present with AF-related symptoms [e.g. general practitioner (GP), the emergency department (ED), or a cardiology clinic]. Our access to non-local GP and ED records was limited; therefore, we could not obtain reliable data on clinical AF without significantly reducing sample size. Nonetheless, this is an important area to investigate and should be considered as part of future prospective trials assessing the significance of POAF in the long term.

Given the mixed evidence for both provoked AF and subclinical, device-detected AF, there is a clear need to identify more accurate predictors to identify those at higher risk of developing recurrent AF, to develop a unified approach to timing and duration of postoperative ambulatory ECG monitoring, and to establish the best treatment of POAF. There are ongoing prospective trials assessing the effect of OAC in patients with POAF after cardiac surgery (NCT04045665, “PACeS” study) (32) or non-cardiac surgery (NCT03968393, “Aspire-AF” study) (33). PACeS is a prospective, multicentre, open-label, randomised trial comparing OAC with no OAC (in addition to usual anti-platelet therapy) for patients with POAF after CABG and is estimated to complete in June 2025. Hopefully, prospective studies such as these will help shape future guidelines.

In the devices arm of the study, we found that surgery involving the AV was associated with a higher incidence of a device being inserted within 30 days of the operation compared with CABG-only of MV surgery [CABG-only, 44/6,470 (0.68%); AV surgery ± CABG, 115/3,244 (3.5%); MV surgery ± CABG, 18/1,095 (1.64%); *p* < 0.0001, Figure 2]. Our results are consistent with previous findings but show lower rates compared with historical trials, which may reflect improvement in surgical techniques and technology over time (19, 34).

Limitations of our study include the retrospective observational design, meaning that we can only comment on associations and not draw conclusions about causality. The sample sizes were small, in particular for the recurrent AF arm of the study, as many patients had their device checks performed at other centres, and these data were not accessible to us. Although a significant association was found with POAF and recurrent AF, the confidence intervals were wide, so the true effect size is not precisely established in this study. The requirement for a cardiac device also limits the wider application of our findings and may have induced a selection bias, particularly by reducing the number of patients undergoing CABG and MV surgery, as there was a lower incidence of device insertion/postoperative conduction disease in these patients. Finally, our study is single-centred limiting its generalisability as our results may be influenced by surgical techniques and demographics of the local population.

## Data Availability

The raw data supporting the conclusions of this article will be made available by the authors, without undue reservation.
